# 

**Published:** 1917-05-19

**Authors:** 


### Hospital and Institutional Results.

**Mount Vernon Hospital for Consumption.**—The Hospital at Northwood is reserved for those who can contribute in respect of the cost of their treatment as in-patients, and towards the expenditure for 1916—namely, £12,774—patients paid, or had paid for them, £5,858. The out-patient department in Fitzroy Square dealt with 13,354 attendances last year. The annual report contains only thirty pages.

**Manchester Royal Infirmary.**—The report is issued in the customary form. The Royal Red Cross (First Class) has been awarded to the lady superintendent, Miss M. E. Sparshott and four sisters, and three nurses received the Royal Red Cross (Second Class), while five sisters and two nurses have been mentioned in dispatches. A side ward of one of the surgical units has been named "The Walter G. Carnt Ward" in memory of the late general superintendent and secretary. The total income for the year was £59,758, and the expenditure £64,326. The endowment fund, by means of special legacies and donations, was increased by £6,750.

**Royal Devon and Exeter Hospital, Exeter.**—The number of patients kept waiting for admission during the past year has not been quite so high as in previous years, chiefly by reason of the fact that subscribers have followed the suggestion made last year by making inquiries beforehand as to available beds. The total income (including £5,945 legacies) was £15,207, and the total income £14,820. The ordinary expenditure was £785 more than in the previous year.

**Edinburgh Royal Infirmary.**—This report contains 264 pages. The governors are asked to agree to the suspension of the rule which requires all physicians and surgeons to retire at the age of sixty-five, this suspension to continue for the duration of the war. The house steward's department cost £33,161, as against £26,549 in the previous year.

**Miller Hospital, Greenwich.**—The notice convening the annual meeting includes a copy of the accounts for 1916. Although only £100 was received under legacies the total income exceeded the expenditure by £711, a result which was not attained by many hospitals last year.

**Essex County Hospital.**—The report is greatly abbreviated, and no list of contributors is issued. The total income for 1916 was £12,666, including £493 legacies, and the total expenditure was £12,909.

### "SHELL" BRAND FLOOR-POLISH.

Members of the nursing staffs in hospitals, infirmaries, and kindred institutions take pride in a well-polished floor. To achieve success it is necessary to select with care the floor-polish used, and it is well to know, in this connection, the "Shell" brand polish supplied by Messrs. Archibald H. Hamilton and Co., of Possilpark, Glasgow, who were many years ahead in placing this polish on the market. "Shell" polish has stood the test of time and competition satisfactorily, and it claims to hold the premier position. Weighted brushes, which have many excellences, are also supplied by Messrs. Archibald H. Hamilton and Co.

### Matrons' and Sisters' Appointments.

Finchley Cottage Hospital.—Miss C. Maple has been appointed matron. She was trained at Kensington Infirmary, and has since been staff nurse at the Central London Nose, Throat, and Ear Hospital, Gray's Inn Road, at Charing Cross Hospital, and at the Finchley Cottage Hospital.

City and District Infirmary, York.—Miss Mabel Senior Nuttall has been appointed superintendent nurse to the institution, where she was trained, and has held successively the posts of charge nurse and head night nurse.

Isolation Hospital, Ferry Bridge, near Weymouth.—Miss Mary E. Hornby has been appointed nurse-matron. She was trained at Bucknall Hospital, Stoke-on-Trent, and has been matron of the Isolation Hospital, Nantyglo, Mon.

Staines Union Institution Infirmary, Stanwell.—Miss Chrissie Bowker has been appointed superintendent nurse. She was trained at St. George's Infirmary, Fulham Road, has held the post of temporary superintendent nurse at several Poor-Law infirmaries, and is at present night sister at West Ham Union Sick Home, Forest Gate.

Beckett Hospital, Barnsley.—Miss M. L. Slee has been appointed sister. She was trained at the Royal Infirmary, Halifax, and has had experience in private and district work and military nursing.

Bermondsey Military Hospital, Ladywell.—Miss Ellen O'Hare, Miss Elizabeth Rose, and Miss Margaret Jones have been appointed sisters. Miss O'Hare was trained at the Bradford Royal Infirmary, Miss Rose at the Lambeth Infirmary, and Miss Jones at the Royal Victoria and West Hants Hospital, Boscombe.

City and County of Perth Royal Infirmary.—Miss Jean A. Cobban has been appointed sister. She was trained at the infirmary, Falkirk, and has been sister at the Belvidere Fever Hospital, Glasgow, and at the Morningfield Hospital, Aberdeen.

Prescot Union Infirmary, Lancs.—Miss Jane Jackson, Miss Ethel Wright, and Miss Minnie Millington have been appointed ward sisters at this institution, where they were all trained.

Red Cross Hospital, Sutton, Surrey.—Miss Annie Jameson has been appointed sister. She was trained at Dundee Royal Infirmary, and has since been theatre sister at Glasgow Eye Infirmary and assistant matron at Bangour Hospital. As a member of the Q.A.I.M.N.S.R. she was theatre sister at Croydon War Hospital. She was theatre sister at the Ulster Volunteer Force Hospital, Pau, Basses Pyrenees.

Sanatorium, New Road, Blackpool.—Mrs. E. E. Bennett (*née* Coupe) has been appointed sister. She was trained at Hunslet Infirmary, Leeds, and has since held the following appointments: Sister-in-charge of Westhulme Sanatorium, Oldham; staff nurse at Horton County of London War Hospital, Epsom; sister at Downs Sanatorium, Sutton; and night sister at City of London Hospital for Diseases of the Chest, Victoria Park.

Walthamstow General Hospital.—Miss Elizabeth Frith has been appointed sister. She was trained at Wolverhampton and Staffordshire General Hospital.

**Note.—Particulars of Appointments for insertion in this column will be welcomed.**

### Personalia.

Mr. C. F. Ratcliffe, a nephew of Colonel Brotherton, has accepted the presidency of the Leeds Maternity Hospital.

### Passing Events and Topics.

### M.A.B.'s Provision for Tuberculous Soldiers.

The Metropolitan Asylums Board is making arrangements whereby 150 beds may be placed at the disposal of the authorities for the accommodation of soldiers suffering from advanced tuberculosis.

### London School of Economics.

The time-table for the summer term of the London School of Economics and Political Science contains particulars of an excellent list of lectures on a variety of subjects, many of them of interest to institutional workers.

### British Hospitals Association.

The report of the Council of the British Hospitals Association records that Viscount Sandhurst, P.C., the treasurer of St. Bartholomew's Hospital, has accepted the office of president. The chief feature of the programme of the Association—the annual congress, has not been held since the commencement of the war.

### Theatrical Garden Party.

The committee announce that that joyous function, the Theatrical Garden Party, will be held in the Royal Hospital Grounds, Chelsea, some time in July. Mr. Gerald du Maurier and Mr. Anslow J. Austin, Honorary Organisers, have been compelled to seek a larger site than the Botanic Gardens owing to the vastly increased number of attractions this year.

### "Printers' Pie."

This annual favourite appeared on May 14, the price remaining at 1s., for which amount wonderful value is given.

### Gifts and Bequests.

Mr. T. R. Butcher, of Bath, has left the proceeds of the sale of his house to the Frome Home for Trained Nurses, and the residue of his estate to the British Red Cross Society.

Mr. William Walmsley Simpson, of Winkley, Whalley, colliery proprietor, left £1,000 to the Blackburn and East Lancashire Infirmary and £350 to the Oswaldtwistle Nursing Association.

Mr. John Leigh, of Altrincham, has purchased a large house in Cheshire, with grounds, and converted it into a hospital. The money worth of his magnificent generosity is stated as over £10,000. Mr. Leigh has further undertaken to maintain the institution, after the War, as a permanent home for disabled soldiers.

The late Mrs. Elizabeth Goldsbrough, of Leeming Lane House, Southfield Road, Middlesbrough, left the residue (amounting to about £800) of her estate to be equally divided between the North Riding Infirmary, North Ormesby Hospital, Middlesbrough Workshop for the Blind, and Middlesbrough Nursing Association.

Miss Harriet Inston, of Moseley, has bequeathed £200 to West Bromwich Hospital, £100 each to the Birmingham Skin and Lock Hospital, the Birmingham and Midland Eye Hospital, and the Queen's Hospital; £625 was also left to the General Hospital, Birmingham, for the endowment of a "Harriet and Charlotte Inston" bed, and the residue of the estate to the General Hospital, Skin and Lock Hospital, and General Hospital for Women, all at Birmingham.

Rates of Subscription: Twelve months, 6/6; Six months, 4s.; three months, 2/-, post free to any address in the United Kingdom. Abroad, Twelve months, 8/8; Six months, 5/-; Three months, 2/6, post free. **THE HOSPITAL "Index" to Volume LXI., October 1916 to March 1917, is now ready, price 2½d. per copy, post free.** Address The Manager, The Hospital, "The Hospital" Building, 28/29 Southampton Street, Strand, London, W.C. 2.

